# Prostate cancer in the Arab world: Bibliometric review and research priority recommendations

**DOI:** 10.1080/2090598X.2021.2024984

**Published:** 2022-01-23

**Authors:** Adel Hajj Ali, Hussein Awada, Hasan Nassereldine, Mohammad Zeineddine, Zahy Abdul Sater, Albert El-Hajj, Deborah Mukherji

**Affiliations:** a Faculty of Medicine, American University of Beirut, Beirut, Lebanon; b Global Health Institute, American University of Beirut, Beirut, Lebanon; c Division of Urology, American University of Beirut Medical Center, Beirut, Lebanon; d Division of Hematology-Oncology, American University of Beirut Medical Center, Beirut, Lebanon

**Keywords:** Prostate cancer, Arab region, bibliometrics, screening, oncology, urology

## Abstract

**Objective:**

To conduct a scoping review examining the status of prostate cancer research in Arab countries and systematically map publications across the cancer care pathway. Prostate cancer incidence has been rising in the Arab world and tackling its increasing burden will require evidence-based policies.

**Methods:**

We searched Medline, PubMed and Scopus for peer-reviewed publications related to both our research topic and countries of interest by using controlled vocabulary and keywords. Search results were limited for the period between 2000 and 2020, screened for duplicates, and then included in our study based on pre-specified eligibility criteria. We used a structured data extraction form to extract information related to the article, its methodology, its cancer care pathway, funding status, and authorship.

**Results:**

A total of 4142 publications were retrieved from our search, of which 874 articles remained after applying eligibility criteria. Trends show a steady increase in prostate cancer research in the Arab world. Most studies were focussed on diagnosis and treatment, whereas a lack in studies concerning screening and prevention, as well as epidemiological data, was evident. Most studies were not funded and had no female author. Country gross domestic product and population were positively correlated with its research output. The USA had the highest number of corresponding authors. The majority of Arab-based studies did not involve collaborations with other countries. Most research conducted was basic or clinical studies with a low level of evidence.

**Conclusion:**

Our present review identified significant gaps and limitations in prostate cancer research in Arab countries. Priority areas for research investment have also been highlighted as a first step towards context-specific health policies.

**Abbreviations:**

ASR: age-standardised rate; COVID-19: coronavirus disease 2019; GDP: gross domestic product; HDI: Human Development Index; KSA: Kingdom of Saudi Arabia; UAE: United Arab Emirates

## Introduction

Prostate cancer is a leading cause of cancer-related morbidity and mortality affecting men worldwide. The pathophysiology of prostate cancer in different populations remains poorly understood; established risk factors include advanced age, family history of malignancies, Black race, and certain genetic polymorphisms [1]. Countries with a high Human Development Index (HDI) record higher incidence and mortality rates for prostate cancer compared to those with a low HDI. Recent trends have revealed a steady increase in prostate cancer in Low and Middle Income Countries, which may be due to increased diagnostic testing and changing population demographics [2].

Even though the age-standardised rate (ASR) of prostate cancer remains relatively low in the Arab world compared to Europe and North America, it is predicted that the Arab world will see the highest relative increase in cancer incidence globally. Moreover current data may be inaccurate given deficiencies in access to healthcare, limited cancer registries, and low screening rates in Arab countries [2]. The lower incidence of prostate cancer may also be due to biological differences that are specific to Arab men. For example, several studies have shown that lower rates of prostate cancer persist in populations of Arab immigrants when compared to the non-Arab populations of their host countries [3]. Still, prostate cancer was more common among these Arab immigrants than the Arab locals of their native countries [3]. These studies emphasised how both genetic, environmental, and diagnostic differences are responsible for the discrepancy in prostate cancer incidence and mortality between the Arab countries and the rest of the world.

The Arab world consists of 22 countries, 12 of which are distributed across the Middle East and 10 through different parts of Africa. These countries vary greatly in terms of population, development, socioeconomic stability, and healthcare status [4]. The incidence and mortality of prostate cancer varies in between these countries, partially influenced by the availability and quality of cancer data registries. In many cases cancer incidence and particularly mortality data are derived from modelled estimates rather than verified clinical records [5]. With most countries in the Arab world undergoing demographic transition resulting in population ageing plus improved access to diagnostic testing, regional prostate cancer incidence is increasing, as is the need for context-specific research [6].

Despite the need for anticipatory policies to tackle the expected increase in disease burden, there remains a paucity of accurate epidemiological and demographic data to inform these policies. There remains a relative lack of published medical literature originating from the Arab world. In a study performed in 2016, the average number of medical publications per one million people in the Middle East and North Africa was shown to be equal to a quarter of the world average [7].

To address this relative data poverty and make recommendations for regional prostate cancer research priorities, we aimed to map the landscape of prostate cancer research in the Arab world. To establish the gaps in our knowledge base to be addressed, we assessed methodology and productivity in different areas, with a special focus in the analysis on collaboration, diversity, and the extent of funding for publications. We also reviewed the epidemiological and demographic differences between these countries as we compared trends in research output.

## Methods

A comprehensive search of all prostate cancer publications was carried on PubMed, Medline, and Scopus up to January 2021. A Boolean operator (AND, OR, and NOT) in addition to [ad] was used to conduct the search. The terms used in the search included ‘prostate cancer’, ‘prostate carcinoma’, ‘PSA’, ‘prostate adenocarcinoma’, ‘prostate metastasis’, ‘prostate oncology’, ‘prostate neoplasm’, ‘prostate neoplasm’, ‘prostate malignancy’, ‘prostate nodule’, ‘prostatectomy’, ‘Gleason score’, and ‘prostate biopsy’. The articles were chosen according to the following eligibility criteria: articles published between 2000 and 2020, articles that include at least one author affiliated to an academic institute or research facility in the 22 Arab countries listed below, and the article should be discussing any topic pertaining to prostate cancer. We then reviewed articles to select the appropriate match, remove any duplicates, and identify the funding status, presence or absence of a female author, study design, type of publication, number of citations, each author’s country, and the paper’s research question. Gender-neutral author’s names were hand-searched for accuracy.

The following 22 Arab countries were included: Algeria, Bahrain, Comoros, Djibouti, Egypt, Iraq, Jordan, Kuwait, Lebanon, Libya, Mauritania, Morocco, Oman, Palestine (West Bank and Gaza), Qatar, Kingdom of Saudi Arabia (KSA), Somalia, Sudan, Syria, Tunisia, United Arab Emirates (UAE), and Yemen.

For each country, we obtained the gross domestic product (GDP) and the population size from the World Bank. In addition, the ASR of prostate cancer was obtained in the selected countries [8]. To minimise the bias between Arab countries, we divided the number of publications of each country by its corresponding GDP, population size, and ASR to obtain the number of publications per billion GDP, per million persons, and per ASR respectively.

The analysis was conducted on both Statistical Package for the Social Sciences (SPSS®; SPSS Inc., IBM Corp., Armonk, NY, USA) and R (R Foundation for Statistical Computing, Vienna, Austria) statistical tools. Numerical data are reported as frequency and percentage. The data were analysed by chi-square analysis and an α < 0.05 was considered significant. Graphical data are presented by tables, pie charts, bar graphs, cluster graphs and a heat map.

## Results

### Mapping the characteristics of the publications

**Figure 1.**
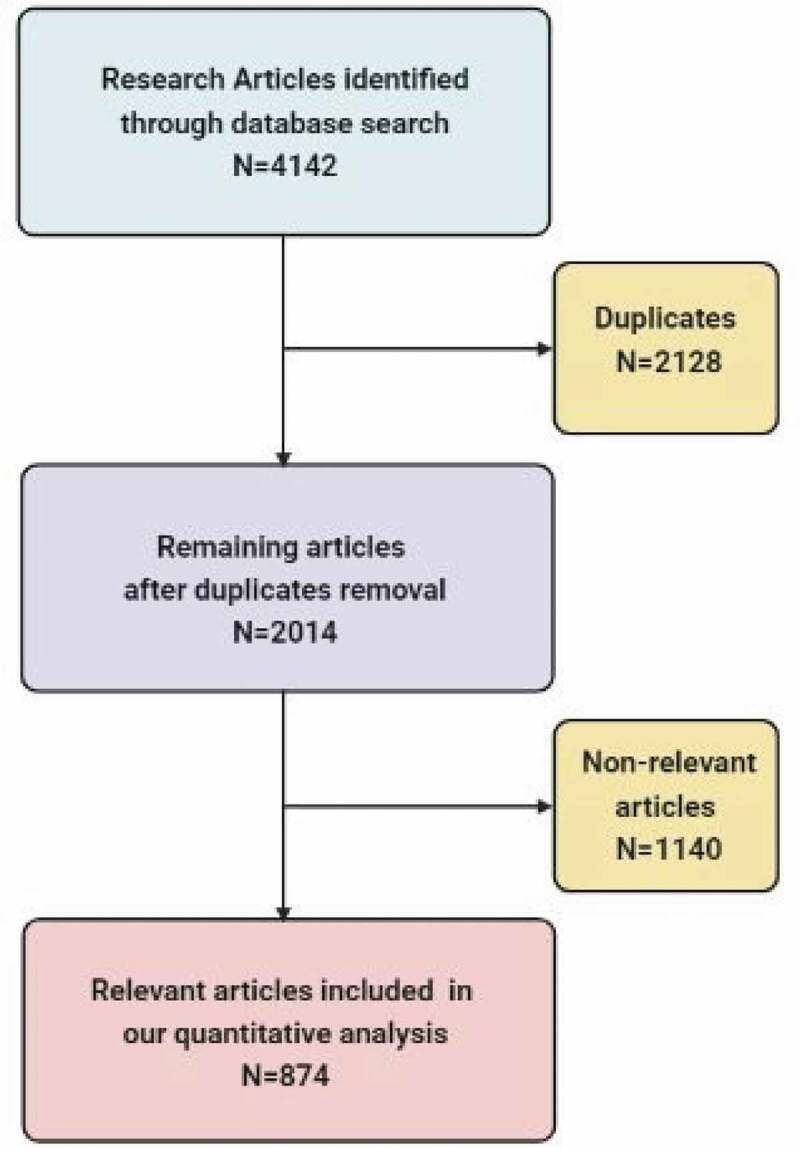
Flow chart of the search results.

From a total of 4142 publications retrieved from the databases, 2128 duplicate articles were excluded; the remaining 2014 articles were assessed by titles and abstracts, of which 874 were eligible for the study and 1129 articles were excluded (Figure 1). Most of the included publications were in English, two articles were in French and one in Italian.

**Figure 2.**
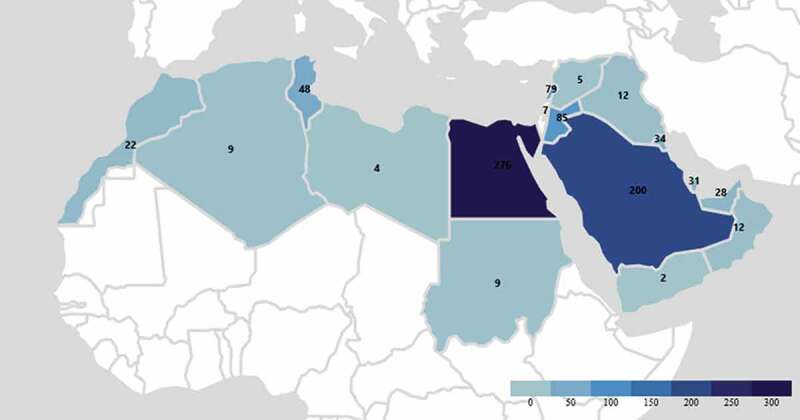
Geographical representation of the articles across the Arab countries.

**Figure 3.**
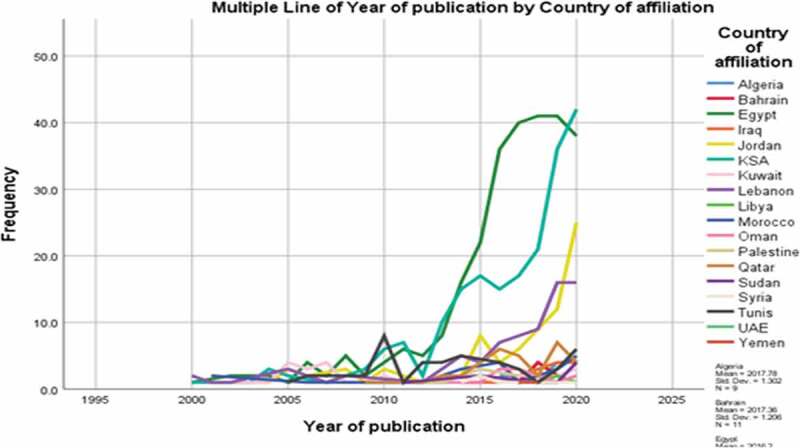
Trends of publications of each Arab country across the years 2000–2020.

Research output in 18 out of 22 Arab countries was related to prostate cancer, with Egypt having the highest number of publications, followed by KSA and Lebanon, while Comoros, Djibouti, Mauritania, and Somalia had no published articles (Figure 2). The trend of publications by country of affiliation across the years (2000–2020) showed a general increase in the yearly total output (mean increase of 17 publications/year) and a notable rise in the outputs of Egypt, KSA, Jordan, and Lebanon (Figure 3). The publications were issued in a wide variety of journals with no journal having >20 (2.3%) publications related to prostate cancer in the Arab countries.

**Figure 4.**
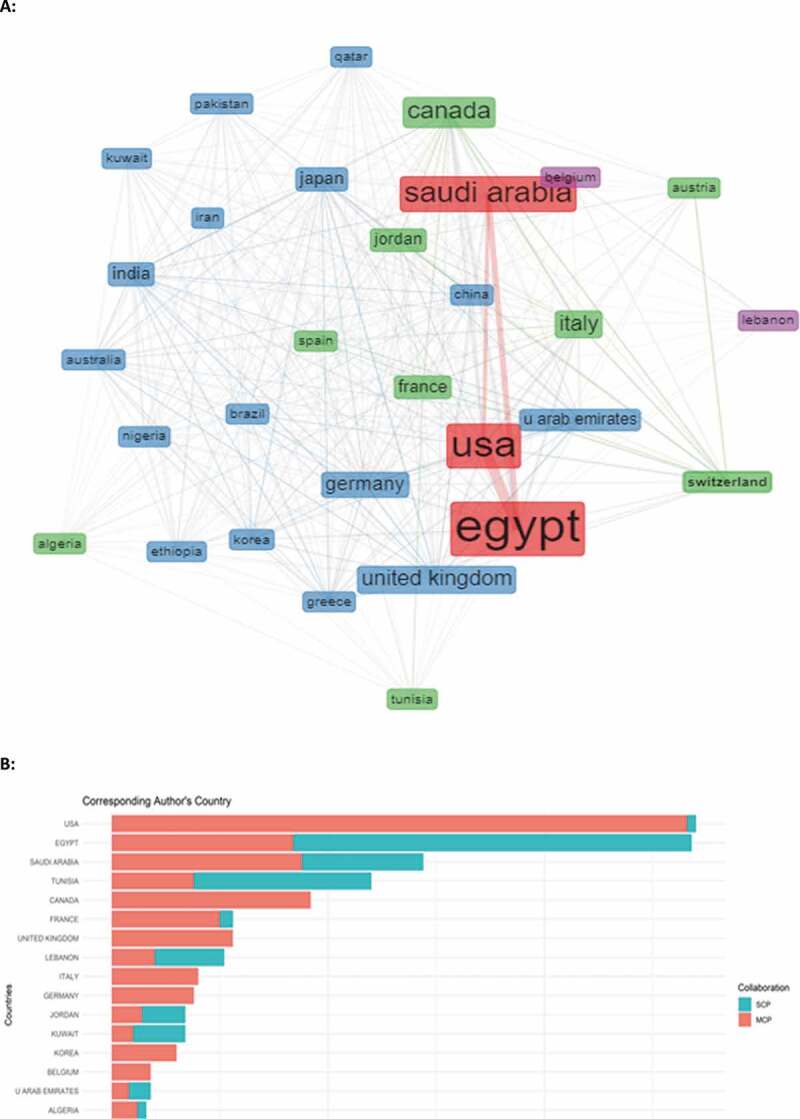
(a) Graphical demonstration of the collaboration among the Arab countries and between the Arab countries and the world. (b) Bar chart showing the country of the corresponding author and its frequency.

Only 37.9% of the total number of articles were funded. Of the funded articles, 39.9% of the funds were from Arab sources, while 60.1% were from non-Arab countries; in addition, 41.6% was from public sources and 58.4% from private sources of funding. Multiple collaborations have taken place among the Arab countries and between the Arab countries and the world on the topic of prostate cancer; however, our analysis sheds light on a strong collaboration between the KSA and the USA, and between Egypt and the USA. Corresponding authors originate mainly from the USA, followed by Egypt, KSA, and Tunisia. In addition, the following countries Egypt, Tunisia, Lebanon, Jordan, Kuwait, and UAE had more single country publications than multiple country publications as opposed to KSA, Algeria, and Bahrain (Figure 4).

### Publications across the cancer care pathway

The articles demonstrated various cancer care pathway topics. In all, 55.8% of the articles covered diagnosis and treatment, followed by 30.8% for risk factors and prognosis, 5.0% for epidemiology, 3.3% for screening and prevention, 1.7% health system studies, 1.7% knowledge and education, 1.3% for palliative care and metastatic diseases, and 0.5% for mental health. Most of the funded articles represented diagnosis and treatment (56.7%) followed by risk factor and prognosis (30.0%).

### Publications and epidemiology

When normalised to population size, Arab countries with relatively small population sizes such as Lebanon, Qatar, Jordan, and Kuwait had the largest output of publications. Next, when adjusting to the GDP of the country, Jordan, Lebanon, and Tunisia had the largest output. Furthermore, a significant positive correlation between the number of publications and the country’s GDP was noticed (*P* = 0.001). Similarly, we adjusted the number of publications to the GDP per capita, which showed a measurable advantage for Egypt.

Our analysis of the studies for the presence of at least one female author among the authors shows a proportion of only 35.3% (*n* = 313). Furthermore, when dissecting the type of studies with at least one female author, we found that 66.1% (*n* = 207) were basic science publications while 33.9% (*n* = 106) were clinical studies reflecting the fact that women remain underrepresented in academic and leadership positions in the region.

### Type of studies

Most of the articles were clinical and biomedical studies (91.6%), while public health studies represented 8.4%. In all, 78.4% of the articles were original research papers, while 18.8% were review articles. As for the study design of the published articles, 26.6% represented basic science research, 18.1% cohort studies, 17.7% descriptive studies, 11.3% quantitative studies, 9.4% case reports, 6.1% case control, 5.3% cross-sectional studies, 4.4% clinical trials, and 1.2% qualitative studies. Analysis of citations showed only four articles that had >1000 citations, 13 articles having between 100 and 1000 citations, and 127 articles having no citations.

## Discussion

Our present search of the main medical bibliographic databases for peer-reviewed publications concerning prostate cancer yielded a mere 874 contributions from the Arab world from the year 2000 to 2020, equivalent to <0.5% of the total prostate cancer research produced worldwide since the beginning of the 21st century. These findings were somewhat comparable to the Arab contribution to the study of other types of neoplasms such as breast cancer (0.8%), suggesting that this scarcity actually pertains to cancer research in general [9].

Fortunately, our present data indicate that the Arab world has been witnessing a continuous growth in prostate cancer research over the last 20 years, with the exception of the period between 2010 and 2012. This period coincided with the height of the ‘Arab Spring’, during which political instabilities and conflict took their toll on medical research by exacerbating the brain drain to the West and shifting funding away from research [4,7,10]. Indeed, ongoing conflict-affected countries in the Arab world including Syria, Yemen and Libya have contributed the least to prostate cancer research among Arab countries as per our search. Similar findings were also found for breast cancer research conducted in conflict-affected Arab countries in the scoping review by Abdul-Khalek et al. [11].

Despite the trend of overall yearly increase in publications, the Arab world still falls of other regions of the world, especially in epidemiological studies, as well as research investigating the clinical role and benefits of screening and prevention. Both of these tracks are fundamental in addressing the rising rates of prostate cancer in the Arab world. Interestingly, our present analysis showed that funding was not a factor behind the discrepancy in research output across the cancer care pathways. This suggests that other factors may possibly be behind this variation, such as the lack of research capacity and research collaboration. The variation may also be due to the lack of global consensus for screening and the relatively high survival rates of prostate cancer [12]. The deficits in these studies serve as a warning signal because of the importance of epidemiological data as the base from which evidence-based public policies are informed, and the role of clinical screening studies that ‘clinically’ guide these policies [13].

Funding for medical research remains one of main factors hindering its progression in the Arab world, with the number of Arab-based institutions funding cancer research, in particular, being a slim minority compared to the rest of the world [7,14]. Our present data further showed the existence of a significant disparity in funding or prostate cancer research among the Arab countries. It also revealed how public health research on one hand, and the clinical branch of medical research on the other, were significantly underfunded. While we emphasise the importance of basic research, financing clinical medical and public health research is equally important as they target the human population and health systems in an effort to reduce the burden of preventable morbidity and mortality [15].

Our present results further showed that the research output among the Arab countries had a significantly positive correlation with national GDP, and that the correlation was even stronger when adding the population size into the equation. While these findings conform well with the literature, most of the Arab countries do not rank high when it comes to GDP [16–18]. This limits the capabilities of the lower-siding countries to allocate funds for research. Unfortunately, because of the ongoing regional conflicts, coronavirus disease 2019 (COVID-19) induced financial constraints and longstanding economic instabilities affecting some Arab regions, efforts to allocate governmental funds towards medical and public health research may not be worthwhile in some countries [4,7,19]. Instead, efforts in these countries must be directed towards bypassing national limitations by establishing regional and international collaborations with other Arab and non-Arab institutions in order to increase the quantity and quality of their research output [20,21]. Mutually beneficial collaborations can be set up, in which the concerned parties complement each other’s deficits in funding, skills, expertise or other resources. Our present results show that such collaborations do currently exist but only to very limited extents. This is according to our findings that most foreign-based publications (according to corresponding authors) from the USA, Canada, France, UK, Italy and Germany involve multiple country contributions, whereas the majority of Arab-based publications utilise contributions from only a single country. This may suggest that collaborations are primarily initiated by the non-Arab rather than the Arab countries. Furthermore, six countries among the top 10 contributors are non-Arab. These revelations further assert the lack of initiatives on behalf of the Arab countries to both conduct prostate cancer research, as well as establish collaborations with the rest of world in an attempt to overcome any research obstacles and bypass capacity limits.

Finally, female investigators remain underrepresented in urology-related research in the Arab world despite the worldwide trend towards increasing female involvement in the field of urology [22]. Cultural and stigmatic effects persist up to this day, with our present findings showing that the significant majority of women scientists contribute to laboratory-based research instead of clinical research. Women comprise ~70% of global healthcare workers and improving gender equity and diversity in research teams can accelerate the growth in prostate research across the Arab world. Techniques to establish the gender of authors are not 100% accurate so this should be mentioned as a limitation of our analysis. Our main findings and recommendations are summarised in Table 1.

**Table 1.** Summary of findings and recommendations

Finding	Recommendation
Lack of studies focussing on screening and prevention of prostate cancer	Encouraging these categories of prostate cancer research among others while also allotting a specific part of the national research funds for their needs.
Shortage in epidemiological, public health and clinical studies providing high levels of evidence to inform clinical practice	
Lack of financial support for prostate cancer research	Increasing initiatives among Arab institutes towards establishing regional and international collaborations through which they can bypass obstacles and limitations in research capacities.
Insufficient collaboration with regional and international institutes	
Low female participation in urology related research	Breaking down social stigmas by recruiting female researchers and asserting their importance in increasing research capacity and filling the gaps in manpower.

To the best of our knowledge, this is the first bibliometric analysis to study the Arab world prostate cancer research output. However, this study has some additional limitations. First, the study results might not represent the true scale and intent of research of some of the Arab countries because of the coinciding regional conflicts and publication barriers. Second, bibliometric studies, unlike systematic reviews, focus on the methodologies and designs of research papers instead of the significance of their results. Analysis of impact factor/citations of each paper was not possible due to the volume and time interval of publications reviewed. Third, like all the other bibliometric studies, we were reliant on the indexing of the databases accessed.

## Conclusion

Prostate cancer incidence is increasing in the Arab world with expected increases in morbidity, mortality, and healthcare costs. Improving impactful research activity is a key factor in improving regional health policy and cancer control. Although there is a general increase in research output in the Arab world, we have noted significant limitations to be urgently addressed. Priority areas for investment include epidemiology, screening and prevention, high-quality clinical research, and initiatives to promote diverse regional and international collaborations.
